# Mapping Environmental Inequalities Relevant for Health for Informing Urban Planning Interventions—A Case Study in the City of Dortmund, Germany

**DOI:** 10.3390/ijerph13070711

**Published:** 2016-07-13

**Authors:** Johannes Flacke, Steffen Andreas Schüle, Heike Köckler, Gabriele Bolte

**Affiliations:** 1 Faculty of Geo-Information Science and Earth Observation (ITC), University of Twente, P.O. Box 6, Enschede 7500 AE, The Netherlands; 2 Department of Social Epidemiology, Institute for Public Health and Nursing Research, University of Bremen, Grazer Str. 4, Bremen 28359, Germany; steffen.schuele@uni-bremen.de (S.A.S.); gabriele.bolte@uni-bremen.de (G.B.); 3 Department of Community Health, Hochschule für Gesundheit, Gesundheitscampus 6–8, Bochum 44801, Germany; heike.koeckler@hs-gesundheit.de

**Keywords:** environmental inequalities, health determinants, health equity indicators, urban planning, neighborhood, environmental justice, health inequalities, built environment, socioeconomic position

## Abstract

Spatial differences in urban environmental conditions contribute to health inequalities within cities. The purpose of the paper is to map environmental inequalities relevant for health in the City of Dortmund, Germany, in order to identify needs for planning interventions. We develop suitable indicators for mapping socioeconomically-driven environmental inequalities at the neighborhood level based on published scientific evidence and inputs from local stakeholders. Relationships between socioeconomic and environmental indicators at the level of 170 neighborhoods were analyzed continuously with Spearman rank correlation coefficients and categorically applying chi-squared tests. Reclassified socioeconomic and environmental indicators were then mapped at the neighborhood level in order to determine multiple environmental burdens and hotspots of environmental inequalities related to health. Results show that the majority of environmental indicators correlate significantly, leading to multiple environmental burdens in specific neighborhoods. Some of these neighborhoods also have significantly larger proportions of inhabitants of a lower socioeconomic position indicating hotspots of environmental inequalities. Suitable planning interventions mainly comprise transport planning and green space management. In the conclusions, we discuss how the analysis can be used to improve state of the art planning instruments, such as clean air action planning or noise reduction planning towards the consideration of the vulnerability of the population.

## 1. Introduction

The World Health Organization (WHO) and UN Habitat (United Nations Human Settlements Programme) acknowledge in their 2010 report The Hidden Cities that “Where in a city you live and how the city is governed can determine whether or not one benefits from city living” [1]. Such spatial differences in urban areas resulting from environmental conditions in which people grow, live, work and age contribute to health inequalities within cities [2].

In the literature, two explanations for spatial health inequalities are discussed. First, at the level of aggregated data, health differences between populations of different areas can be attributed to differences in the composition of neighborhood residents relating to individual socioeconomic status or health-related behaviors (compositional effect). Second, spatial variations in health outcomes are attributed to the characteristics of the local built and social environment (contextual effect) [3,4,5,6,7]. Relevant characteristics of the built environment comprise both environmental burdens, e.g., air quality and noise, and environmental benefits, e.g., access to parks and services. There is evidence that both aggregated socioeconomic characteristics of neighborhoods and built environmental factors have an independent effect on individual health outcomes [8,9,10,11,12,13].

Conceptual models that describe social inequalities in health outcomes [14] are descriptive and useful for identifying determinants affecting the health of individuals. If these models also include factors describing the built environment, they can be used to ascertain whether specific groups of society are facing a disproportionate share of environmental burdens compared to other groups. “The term ‘disproportionate’ means that the magnitude of health and environmental impacts is greater for a given community or population as compared to a reference counterpart, such as a comparable community or the area surrounding the target community” [15] (p. 171f). The notion of disproportionate share of burdens is also applied regularly in environmental justice analyses [16,17]. According to Walker [18] (p. 40f), an environmental justice analysis is based on the concepts of inequality as a descriptive term and justice as a normative term. In this paper, we focus solely on the (descriptive) identification of environmental inequalities relevant for health.

Addressing environmental health inequalities has become an important issue in recent years, particularly in policies relating to urban development and environmental planning, because suitable planning interventions can affect health through impacts on the context in which individuals live [19,20]. Gelormino et al. [21] identified the built environment as an important policy domain having an impact on health inequalities, although it is rather seldom addressed. Local plans and programs, such as air quality plans, noise protection measures and the development of urban green infrastructure, are typical examples of suitable planning-related interventions. Bambra et al. [22] found evidence that urban planning interventions, particularly in the housing and transport sectors, e.g., traffic calming schemes, promotion of walking and cycling and changes in housing infrastructure, may diminish social gradients in health. Accordingly, Braubach and Grant [23] call for an integrated approach involving urban planners, public and environmental health professionals, other relevant sectors and administrations at different levels in order to improve physical, mental and social well-being by means of urban planning.

Recent international programs and projects, such as the current phase of the WHO Healthy Urban Planning Initiative [20] and the Healthy Urban Development Checklist [24], have picked up these issues striving for a better integration of planning and health in order to mitigate health inequalities. However, in practice, Abernethy [25] observes “siloed problem solving attempts” limiting a successful collaboration between various groups in order to solve environmental health-related problems. De Leeuw et al. [26] report barriers to integrating health plans with land use or other local governmental plans, including lack of collaboration across sectors, workforce capacity issues and the complexity of council planning requirements. Other factors limiting the collaboration between public health and urban planning, as observed in this project, are of a terminological, as well as a methodological nature. For instance, the population-based approach of public health studies, sometimes neglecting contextual, location-related impacts, contrasts with the spatial, location-based approach of urban planning. 

A promising approach to addressing urban health inequalities is the development and application of urban health equity indicators. Friel et al. [27] (p. 870) claim that a comprehensive range of indicators is needed to address social and environmental determinants of health equity. In its 1999 report on environmental health indicators [28], the WHO demanded the development of such indicators in order to support and monitor policies on environment and health at all levels, though at that time, it did not explicitly mention health inequalities. Two subsequent WHO reports [2,29] explicitly postulate the development of health equity indicators, particularly for urban areas in the Global South, to monitor social determinants of health and to develop suitable interventions. Fairburn and Smith [30] developed an indicator-based approach including health inequalities from an integrated perspective of environmental justice and quality of life for the region of South Yorkshire. The need for information on health disparities for small geographic areas is stressed by Rothenberg et al. [31], who developed an urban health index for census tracts for the City of Atlanta, U.S., based on indicators for seven health determinants. Corburn and Cohen [1] discuss how the development of such indicators can act as an instrument for urban health governance, as it helps to identify relevant health policy issues, to generate standards for health equity issues and to improve public accountability and transparency. In addition to their function of assessing and monitoring health inequalities, such urban health equity indicators may also be used to support local stakeholders in identifying planning interventions addressing health inequalities [32].

This paper aims to identify socioeconomically-driven environmental inequalities relevant for health in order to determine the available options for planning interventions from a city-wide perspective by means of neighborhood indicators. To illustrate the applicability of the approach, we calculated all neighborhood indicators for the city of Dortmund, Germany, as the case study area, representing typical medium- to large-sized cities facing significant differences in living and environmental quality, as well as socio-structural composition. Building upon the Spatial Urban Health Equity Indicators (SUHEI) framework [33], we first developed suitable indicators, reflecting problematic environmental health-related conditions in Dortmund. We then applied statistical correlation analysis in order to determine associations between socioeconomic indicators and environmental burdens and resources at the neighborhood level in order to identify health-related inequalities between 170 neighborhoods of Dortmund. The results could assist with the targeting of appropriate planning-related interventions. Our hypothesis is that in Dortmund, people living in neighborhoods with a low socioeconomic position are disproportionally more exposed to negative environmental conditions affecting their health than people living in neighborhoods with a higher socioeconomic position.

## 2. Materials and Methods

The Spatial Urban Health Equity Indicators (SUHEI) framework [33] allows us to map exposure to environmental factors affecting health determined by various drivers and pressures. The purpose of the model is to map areas showing a disproportionate exposure of certain socioeconomic groups to environmental burdens in order to identify appropriate planning interventions. Building upon the ideas of Morris et al. [34], who added social context variables to the Driving force-Pressure-State-Exposure-Effect-Action (DPSEEA) framework [28] characterizing the population, the SUHEI framework combines elements of environmental health-related cause-effect indicator frameworks [35] with common (environmental) health equity models [14]. 

**Figure 1:**
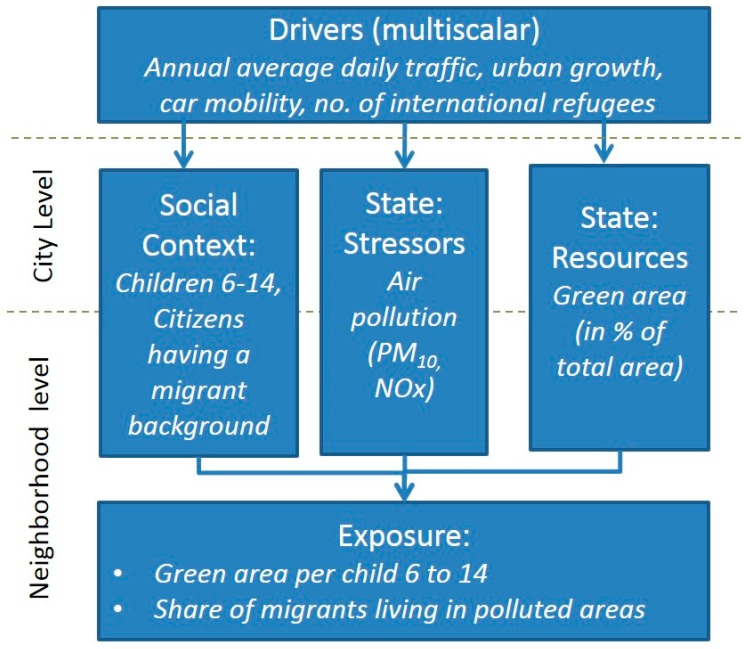
Spatial Urban Health Equity Indicator Framework (SUHEI) (after [33]).

The SUHEI framework distinguishes driver, state and exposure indicators of determinants of health, captured on multiple spatial scales (Figure 1). Drivers, appearing on various scales from national to sub-local, represent factors that motivate and push the environmental or social processes involved, such as increasing traffic density, public spending or urban development. State indicators, reflecting the current status, map concrete environmental stressors and resources (burdens and benefits), as well as relevant social context variables, both at the city and neighborhood level. Finally, exposure indicators relate the environmental state to social context indicators in order to spatially target health inequalities, e.g., neighborhoods where a high level of noise-related impacts matches with a disproportionately higher share of unemployed or deprived inhabitants. By combining multiple environmental burdens and benefits, cumulative environmental impacts may also be related to social context factors [36]. Exposure indicators, mapped at the neighborhood level, are intended to guide planners in identifying hotspots where specific action needs to be taken, while state and driver indicators help to define what kind of measures are to be taken.

The purpose of the indicator model is to examine geographic patterns and identify hotspots of environmental socioeconomically-driven health inequalities. Therefore, state, social context and exposure indicators included in the model are measured in a spatially-explicit manner on a neighborhood scale. In this context, neighborhoods are understood as determining the availability of and access to health-relevant resources in a geographically-defined area [3]. The mapping of indicators at the neighborhood level is indispensable for two reasons. Firstly, neighborhoods are a typical size for health-related urban planning interventions. Secondly, taking into account that environmental justice issues are very sensitive to scale [37], neighborhoods are sufficiently small in size and homogeneous in terms of their socio-spatial structure to allow us to derive evidence of disproportionate impacts.

The city of Dortmund is divided into 170 neighborhoods ranging from 0.1 km^2^ to 6.2 km^2^ in size. These neighborhoods can be distinguished into urban and suburban neighborhoods, based on a number of socio-demographic, economic, mobility and housing variables. A cluster analysis carried out by the city administration [38] distinguishes 3 different types of urban clusters containing in total 80 neighborhoods (two clusters of around 150 inhabitants/ha population density, one cluster showing a population density of 350 inhabitants/ha) and 2 types of suburban clusters with a total of 90 neighborhoods both having a population density of about 80–90 inhabitants per ha.

### 2.1. Case Study Area

The city of Dortmund (280 km^2^) is located in the western part of Germany in the former coal mining and steel-producing, highly urbanized region of the Ruhr. The city is home to nearly 600,000 inhabitants [39]. As a result of the economic boost in the middle of the 20th century, Dortmund attracted a large number of migrant workers. Since the 1980s, Dortmund has been through and continues to go through a long-lasting economic transformation due to the closure of coal mines and steel production companies, resulting in a high unemployment rate (12.5%, in Germany 6.7% (2014)) [39,40]. This ongoing transformation process towards a business, trade and service-oriented local economy has resulted in a highly fragmented city revealing large socio-economic disparities.

The city is an archetypical example of many medium- to large-sized cities in Germany facing significant differences in living and environmental quality, as well as the socio-structural composition of its population after going through a long-lasting phase of socio-economic change. The outcomes of this process are urban land use patterns exhibiting a close proximity of residential, industrial and commercial structures, which may eventually bring benefits of increasing vitality and livability of the neighborhood, but are on the other hand typically associated with negative environmental impacts [41]. Dortmund is characterized by strong social and ethnic segregation [42]. The city’s divide into rather disadvantaged neighborhoods in the north and better off neighborhoods in the south is typical for the entire Ruhr region. Finally, Dortmund reveals significant differences in health outcomes. The average age at death in the districts of Dortmund in 2011 ranges from 66.3 years in the Nordstadt district to 76.3 years in the Hombruch district [43].

### 2.2. Dataset and Methods

In order to obtain an accurate comparison between neighborhoods, all indicators used in this study are mapped as proportions. Environmental state indicators are measured as impacted area as a percentage of the total neighborhood area, while social context indicators are calculated as the number of people showing certain attributes as a percentage of the total population in the neighborhood (Supplementary Materials, Table S1). Taking the entire area of the neighborhood into account acknowledges the fact that the inhabitants also make use of the non-built-up areas, such as parks, roads, public spaces, e.g., for walking, recreation and working, and they are also exposed to environmental burdens in these areas. The focus on neighborhoods as spatial units of the analysis accords with the availability of socio-economic data acknowledging that this is not necessarily equal to the area of environmental impacts. In order to indicate the need for intervention, environmental indicators are calculated as the proportion of areas where the level of detrimental environmental factors exceeds defined threshold values. The study makes use of the datasets as given in Table 1 and Table 2 to calculate the selected indicators. The GIS and census data used are provided, unless otherwise noted, by the City of Dortmund. All census data of socio-economic variables are measured at the neighborhood level. All indicators are calculated using ArcGIS 10.3 (Redlands, CA, USA).

Spearman rank correlation coefficients between socioeconomic neighborhood variables were calculated in order to identify a representative indicator describing neighborhood socioeconomic position. Relationships between neighborhood socioeconomic position and environmental variables were analyzed on a continuous scale with Spearman rank correlation coefficients. Finally, quartiles of neighborhood socioeconomic variables and environmental variables were generated (Supplementary Materials, Table S2). Relationships between categorical socioeconomic and environmental variables were analyzed with chi-squared tests. All statistical analyses were performed using SAS statistical software package Version 9.4 (SAS Institute, Cary, NC, USA). In order to map cumulative environmental burdens, categorical environmental variables categorized in quartiles from 1 (low) to 4 (very high) were added. In doing so, all indicators were weighted equally. The indicator green areas indicating an environmental benefit were added in reverse order. 

### 2.3. Indicator Development

The selection of indicators for mapping socioeconomically-driven environmental health inequalities in Dortmund was problem-driven and context-specific following a deductive indicator development approach [47]. The selection was guided by recent environmental health studies, theories and conceptual models, as well as the input of stakeholders from the case study area. Selection criteria were the indicators’ relevance for environmental health inequalities, their recognition as a health problem by local stakeholders and the possibility of their being influenced by urban planning.

The WHO [48] names physical activity, social impacts, air pollution, noise exposure and unintentional injuries as the main determinants of health in urban settings. From these, the authors identify physical activity, air pollution and noise impacts as the main aspects that may be influenced by urban planning. Studies analyzing socioeconomically-driven environmental health inequalities in Germany based on aggregated data often use similar datasets and indicators, partly because they offer an accurate reflection of the particular problems of many German cities, partly because the datasets are often available for municipalities. Lakes et al. [49] used transport-induced noise data for measuring environmental burdens and a vegetation index as an indicator for environmental benefits in Berlin. As social context factors, the same authors used a highly aggregated index, including factors, such as unemployment, social welfare recipients, child poverty and inhabitants under the age of 18 with an immigrant background [50]. Raddatz and Mennis [17] identify higher proportions of foreigners, as well as poor people as the main factors determining environmentally-unjust situations in cities with respect to the location of toxic release facilities. Riedel et al. [51] used two factors, the unemployment rate and mean income, to determine spatial health inequalities in the Ruhr area. 

In order to understand stakeholder preferences and priorities, workshops with local stakeholders were conducted in 2014, in which the main environmental problems, as well as typical social context variables indicating inequalities in the city of Dortmund were identified. Due to the industrial development described above, the built environment, particularly in the more deprived neighborhoods, is characterized by historically-developed land use patterns exhibiting a close proximity of residential areas with other land uses, such as industrial and commercial [52]. Such mixed land use structures, though they may eventually have benefits in terms of increasing the vitality and livability of the neighborhood, are typically associated with negative environmental impacts [41]. Accordingly, noise impacts and limited air quality were identified as the main environmental stressors, while access to good quality green areas for the purposes of recreation, etc., was limited in some parts of the city. With respect to social context variables, the city has, due to its economic history, a significantly higher share of population having a background of migration, compared to the German average. In Germany, social status is still very much associated with citizenship and ethnic background [53,54]. Consequently, indicators, such as the proportion of the population with a background of migration and the proportion of families being welfare recipients, are suitable indicators of the social context of inequalities. The following indicators are therefore used in this study (Table 3).

The noise indicator is calculated as the percentage of the total area showing a noise impact of more than 55 db(A) resulting from any of the 5 noise sources included in this study (airport, tram, train, cars, industry). Various studies assume a significant health impact from noise exposure of more than 55 db(A) [55] (pp. 23–25). The actual noise impacts might be even higher in some areas where various noise impacts overlap, producing multiple noise impacts. Air quality in Dortmund is measured using the two most relevant substances, nitrogen dioxide (NO_2_) and particulate matter (PM_10_). As for noise emissions, the indicators are calculated as the percentage of the neighborhood where air pollution is above a certain threshold value. Germany’s Federal Immission Control Act of Germany (Bundes-Immissionsschutzgesetz (BImschG)) defines for both factors a threshold value of 40 μg/m^2^ [56], while other studies suggest from a health perspective much lower values of, e.g., 20 μg/m^3^ for PM_10_, as well as various intermediate values [57]. Due to the fact that modelled air quality parameters in Dortmund for large parts of the entire city range between 21 and 40 μg/m^3^, we have taken a threshold value of 30 μg/m^3^ for NO_2_ and 25 μg/m^3^ for PM_10_ into account based on the distribution of values over the city in order to determine intra-urban differences. Calculating the indicator as a neighborhood-wide average means that in specific locations, e.g., close to main roads, values might even be much higher.

The indicator green areas summarizes the proportion of green areas as a percentage of the entire neighborhood. As such, the indicator focuses on the availability of green areas, while aspects of the accessibility and quality of the area are not taken into account. All green areas and parks, as well as forest areas having a size of more than 1 ha, being the minimal size for having relevant functions, are included in the indicator calculation. Assuming an accessibility of green areas within a 400-m Euclidian distance [58], green areas in adjacent neighborhoods are also taken into account for the indicator calculation.

All social context indicators are calculated as a percentage of the total population in the neighborhood. The two indicators of employment and welfare measure complementary issues of interest, namely the unemployed people plus the welfare subsidies paid for the non-working share of the population, namely elderly, children and young people. Hence, the indicator of socioeconomic disadvantage is an aggregate of both indicators, indicating the total number of inhabitants with welfare needs.

## 3. Results

In the following section, socioeconomically-driven environmental inequalities relevant for health in the neighborhoods of Dortmund will be analyzed based on the indicators discussed above. In doing so, we first analyze associations between social context indicators and their spatial distribution. We then carry out a similar analysis of the environmental indicators, including an analysis of multiple environmental burdens. Finally, we consider the exposure of neighborhoods with significant proportions of socioeconomically-disadvantaged residents and higher levels of environmental burdens, in order to identify spatially the hotspots of environmental health-related inequalities.

### 3.1. Social Context Indicators

Regarding the social context determinants of health, we see a strong positive correlation between all four selected indicators (Table 4). The indicator migration is strongly correlated with both the unemployment and the welfare indicator, confirming the observation that neighborhoods with a higher proportion of residents with a background of migration are more likely to have a higher proportion of socioeconomically-disadvantaged residents. The combined indicator of socioeconomic disadvantage, that integrates working age people with the elderly and children, also correlates strongly with the migration indicator, confirming that a proportion of deprived population exists over all generations. As the combined indicator of socioeconomically disadvantaged is the most comprehensive social context indicator, we use this one in the exposure analysis below.

**Figure 2:**
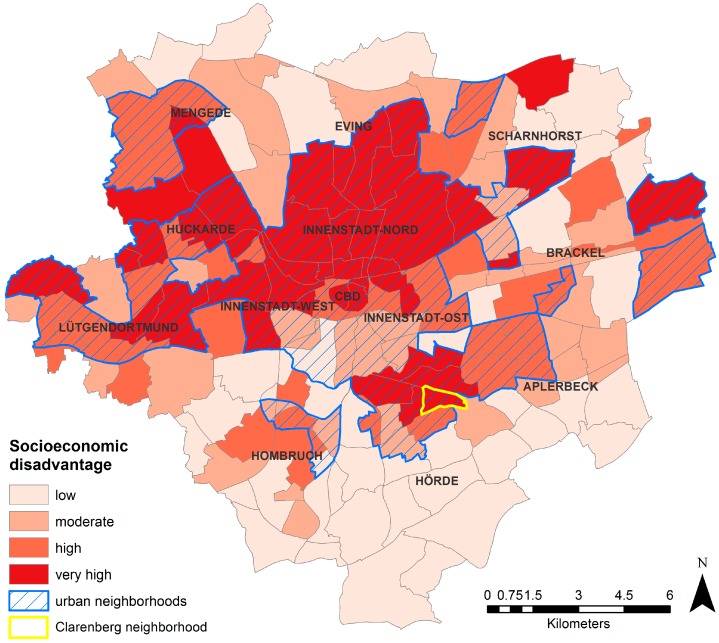
Proportion of socioeconomically-disadvantaged residents per neighborhood.

The spatial distribution of the social context indicator socioeconomically disadvantaged on the basis of neighborhoods (Figure 2) shows that the northern part of Dortmund has a much higher share of socioeconomically-disadvantaged neighborhoods than the southern part. Almost all urban neighborhoods in the northern half, i.e., north of the central business district (CBD), fall into categories of a high to a very high proportion of socioeconomically disadvantaged. South of the CBD, only one cluster of neighborhoods in the district of Hörde is very highly disadvantaged. In contrast, the cluster of urban neighborhoods directly south of the CBD shows a medium to low share of socioeconomically disadvantaged in all neighborhoods.

However, the neighborhood having in absolute terms the highest share of socioeconomically disadvantaged is the neighborhood of Clarenberg in the district Hörde south of the inner city with a share of 36.6 percent of all people receiving some kind of welfare aid. The vast majority of other high scoring neighborhoods with values of around 30% are concentrated in the district Innenstadt Nord north of the CBD. The majority of neighborhoods scoring low in terms of socioeconomically disadvantaged are concentrated in the suburban districts of the urban periphery.

### 3.2. Environmental Indicators

The relationships between the various environmental indicators included in the study show a slightly less homogeneous, but no less significant picture (Table 5). The noise impact indicator correlates moderately positively with the two air quality indicators of PM_10_ and NO_2_, i.e., neighborhoods having a high impact in terms of noise also show significant levels of NO_2_ and PM_10_. This is not particularly surprising, as all three state indicators are at least partially affected by the same driver, namely motorized transport. While four of the five sources of noise combined in the indicator are transport related (car, tram, train, airport), 54.3% of all NO_2_ emissions and 60% of PM_10_ emissions are related to road traffic [45]. All three indicators correlate slightly less and significantly negatively with the green indicator, indicating that neighborhoods having an impact of air, as well as noise pollution also suffer from having a lower share of green areas available.

**Figure 3:**
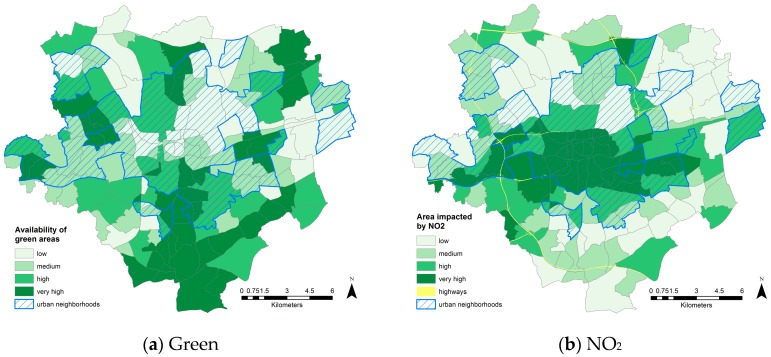

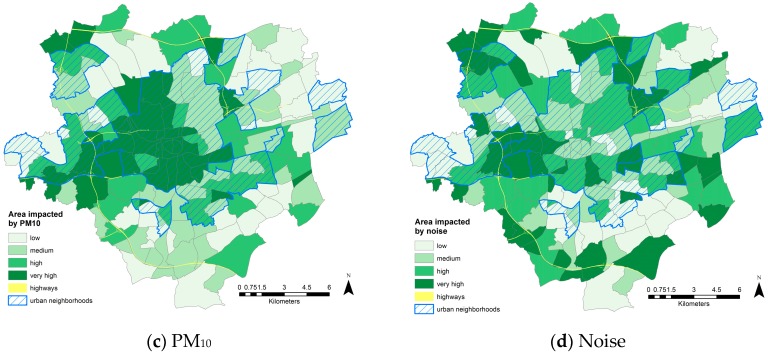
Maps of the four environmental indicators: (**a**) proportion of green areas per neighborhood; (**b**) proportion of area having an annual average NO_2_ level ≥ 30 μg/m³; (**c**) proportion of area having an annual average PM_10_ ≥ 25 μg/m³; (**d**) proportion of area having a noise impact >55 dB(A) L_den_.

The maps shown in Figure 3 exemplify spatial variations concerning the analyzed indicators. Regarding the availability of green areas the most southerly neighborhoods do somewhat better (Figure 3a). In particular, the suburban neighborhoods are much better equipped with green areas, here mainly in the form of urban forests. However, various neighborhoods to the north of the CBD also seem to benefit from a good supply of green areas, e.g., in terms of parks. With respect to NO_2_, the entire central area is very badly affected (Figure 3b), which is very similar to other, comparable German cities, e.g., Berlin [59]. In particular, the neighborhoods around the main highway B1, which cuts through the central areas of Dortmund more or less straight from west to east, are highly impacted. With respect to PM_10_, the situation is similar (Figure 3c), but with a stronger focus in the area to the northwest of the CBD. This area is where the remaining industrial areas in Dortmund are concentrated. Finally, the distribution of noise impacts (Figure 3d) indicates a much more diversified and heterogeneous picture, which is similar to patterns disclosed in other German cities [49]. The industrial neighborhoods west of the CBD stand out as having high noise impacts, as do the neighborhoods along the main highway B1 and A45 in the west (going north to south). Additionally, various urban and suburban neighborhoods reveal high to very high noise impact levels due to local noise sources.

**Figure 4:**
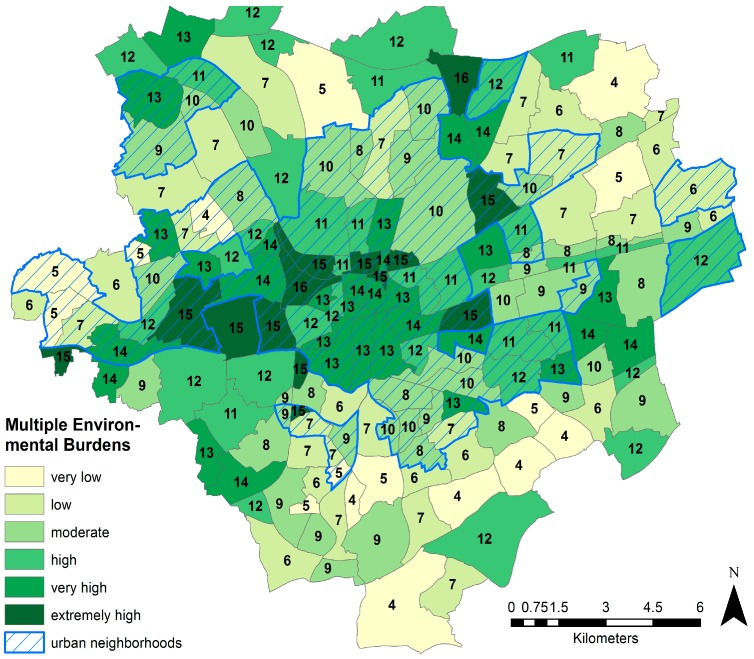
Multiple environmental burdens.

Areas of multiple burdens are neighborhoods where the majority of environmental factors included in the study score high. Figure 4 shows the combination of the four selected environmental indicators. The factor green, being shown above as an environmental benefit, is used here in reverse order indicating a neighborhood of high availability of green with a score of four. The results show that the neighborhoods north of the CBD have the highest levels of multiple environmental burdens, together with the industrialized area west of the city. The neighborhoods scoring highest northeast of the inner city are all areas highly affected by motorized transport.

### 3.3. Exposure: Environmental Inequalities Relevant for Health

In order to identify hotspots of socioeconomically-driven environmental health inequalities, we relate the social context indicator of socioeconomically disadvantaged to the various relevant environmental indicators we have measured (Table 6). The social context indicator correlates moderately negatively with the green indicator, indicating that in areas with a higher share of socioeconomically-disadvantaged residents, less green areas are available. Further, we see a moderately positive correlation between socioeconomically disadvantaged and the two air quality indicators of PM_10_ and NO_2_. All three associations support the hypothesis that the neighborhoods of Dortmund containing higher proportions of socioeconomically-disadvantaged residents suffer from a lower environmental quality compared to the better off areas. Only the noise indicator does not significantly correlate with the social context, indicating that noise is a ubiquitous problem in Dortmund that does not distinguish between the affluent and the disadvantaged.

**Figure 5:**
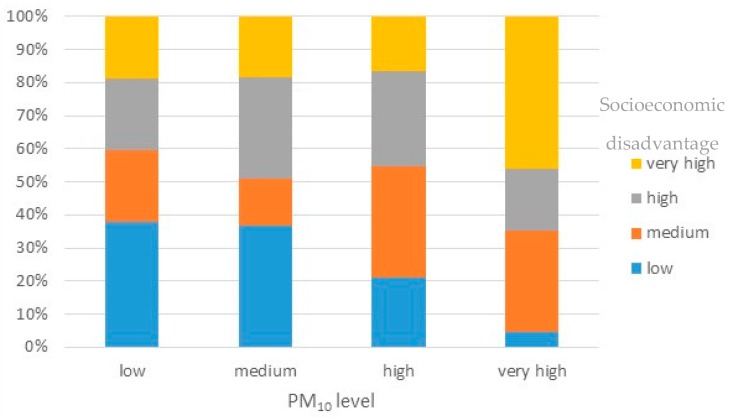
Proportion of socioeconomically-disadvantaged inhabitants per neighborhood in quartiles vs. the proportion of area per neighborhood impacted by PM_10_ above the 25 μg/m^3^ annual mean in quartiles.

The disproportionate supply of environmental goods and bads between neighborhoods of Dortmund having a different socioeconomic profile becomes obvious when considering the spatial pattern of the distribution. Figure 5 relates neighborhoods categorized into four classes according to their share of disadvantaged inhabitants to classes of environmental quality using the PM_10_ indicator as an example. Almost 60% of the neighborhoods having a low PM_10_ level also have a low to medium level of deprivation. In total, 47 neighborhoods of 170 score low to medium for both indicators, PM_10_ and socioeconomically disadvantaged. On the other hand, almost 65% of the neighborhoods showing a very high level of PM_10_ also have a high to very high share of socioeconomically-disadvantaged inhabitants. The difference in proportions is significant (*p*-value < 0.01). Relationships between neighborhood deprivation and NO_2_ and green areas were significant, as well (*p*-value < 0.01). Neighborhoods with a higher proportion of socioeconomically-disadvantaged residents were more exposed to higher NO_2_ levels and had less availability of green space. There was no significant difference in proportions between deprivation and noise.

**Figure 6:**
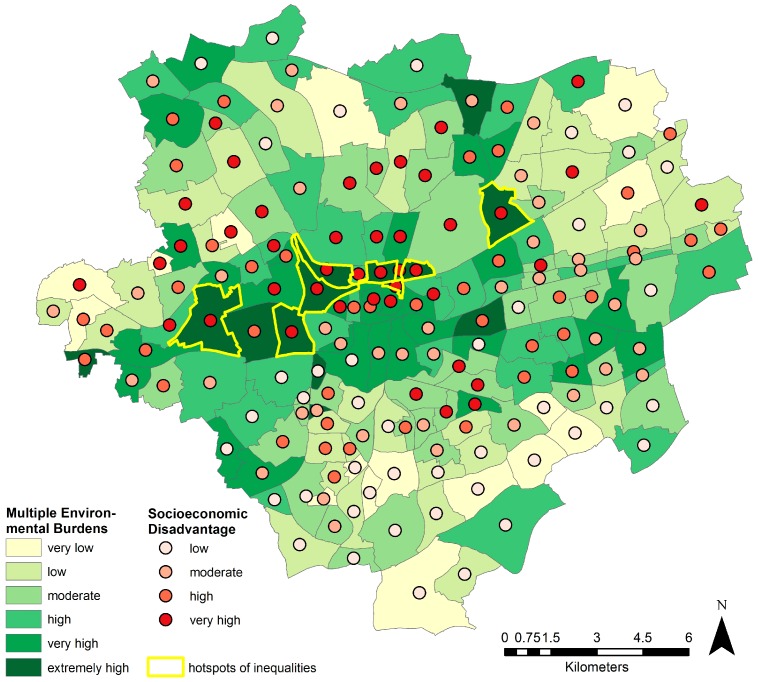
Hotspots of socioeconomically-driven environmental health inequalities: multiple environmental burdens and the proportion of socioeconomically-disadvantaged residents per neighborhood.

The visualization of the exposure in the form of relationships between social context indicators and environment indicators allows the spatial targeting of hotspots of environmental inequalities relevant for health. In Figure 6, the map of multiple environmental burdens is matched with four classes of socioeconomic disadvantage ranging from low to very high (in red dots). Four hotspots of environmental inequalities having the highest proportions of socioeconomically disadvantaged, as well as of cumulative environmental burdens can be identified from this map; the four neighborhoods of the Nordstadt directly north of the CBD, a cluster of neighborhoods west of the inner city, which has already been identified as a rather industrial area, and the neighborhood of Alt-Scharnhorst northeast of the inner city. While other areas scoring highest on multiple burdens show a lower level of deprivation, areas with the highest level of deprivation like the northern parts of the Nordstadt or the District of Hörde southeast of the inner city score modestly in terms of the environmental quality.

## 4. Discussion

The results reveal that certain neighborhoods of Dortmund facing significantly higher impacts from environmental burdens also house significantly larger proportions of inhabitants of lower socioeconomic position. Similar results have been obtained from other studies in comparable cities and regions of Germany. Riedel et al. [51] (p. 88), e.g., determine for the Ruhr region, which Dortmund is a part of, “that individuals with social risks associated with a low education or a low income are more frequently affected by chemico-physical risks”, in this case air pollutants (PM_2.5_) and noise exposure. The geographical pattern of environmental inequalities as derived in this study is comparable to findings from Shrestha et al. [60] for the same city, who locate strong inequalities predominantly in the northern part of the urban core of the city. 

Measuring environmental inequalities relevant for health in the form of indicators represents a useful and easy to replicate approach, especially as working with indicators is common practice in both public health and urban planning [61]. In particular, developing small-scale indicators at a neighborhood level allows the detection of areas of significant inequalities spatially. Moreover, the development of the indicators is based on data that municipalities often collect regularly or that are relatively easy to obtain. Therefore, the approach presented here is relatively easy to replicate. Finally, defining indicators based on existing environmental standards and threshold values, e.g., 55 L_den_ db(A) of noise, allows the application of the model in different political and legislative contexts and offers the possibility of testing different scenarios by varying given standards.

Corburn rightly points to the limitation of indicator-based approaches, saying that “cross sectional measures of single built and social environmental features of urban neighborhoods, tend to ignore the cascading and relational effects of inequalities in urban areas” [61] (p. 23). In a similar manner, it needs to be acknowledged that the indicators used do not necessarily measure all aspects relevant for health. The indicator green areas for instance focusses on the availability of green areas, while the aspects of the accessibility and quality of the area are not taken into account in this analysis. Finally, the study does not include any indicators on the availability of healthcare services and infrastructures, such as hospitals or welfare facilities, which also influence health-related inequalities [62].

Further limitations of the study are that by summarizing several environmental indicators, homogenous impacts on health are assumed per unit, and by using aggregated data for environmental factors, we cannot link levels of exposure to where people live within a neighborhood. Another limitation is the assumption of the spatial homogeneity of the relationships between variables across the study area [63]. Various scholars have recently applied methods like geographically-weighted regression (GWR) to investigate spatial variations in the relationships between predictors, such as environmental or socioeconomic factors, and health outcome variables, such as childhood obesity or physical activity [64,65].

A potential source of uncertainty is the scale of the analysis, particularly the size and assumed homogeneity of the neighborhoods. In order to investigate heterogeneities within our study sample, we performed a correlation analysis separately for urban (*n* = 80) and suburban neighborhoods (*n* = 90). Both analyses yielded very similar results for the correlation as the global analysis; hence, we can assume that spatial heterogeneities are negligible. Additionally, the temporal resolution of the dataset is a potential source of uncertainties for the analysis. While most of the data are from the years 2013 to 2014, the air quality indicators are modelled using base data from 200 to 2012. Hence, the air quality indicators bear a slightly higher level of uncertainty.

The analysis of environmental inequalities relevant for health aimed to identify entry points for urban planning interventions targeting increased urban health equity. The results presented in Section 3 revealed spatial patterns of inequalities across the entire city. These helped to identify hotspots of exposure that might lead to negative health impacts, i.e., neighborhoods with significant proportions of inhabitants of lower socioeconomic positon were exposed to disproportionate environmental burdens. 

The toolbox of urban planning provides different instruments of planning, i.e., specific, more or less judicially formalized and institutionalized plans and procedures that can be implemented in any city. These instruments may address the existing built up area or the development of new infrastructure. As shown in Section 3, several of the identified environmental burdens we identified resulted from transport and industrial activities; others from a lack of access to green areas. Accordingly, transport planning and green space management are relevant sectoral fields of urban planning. Suitable planning instruments addressing existing built-up areas focus on the physical environment based on environmental planning law. The reduction of noise and air emissions is dealt with in instruments that are based on EU legislation, namely the EU noise directive [66] and the EU clean air directive [67]. Both have been implemented in Germany in the Federal Immission Control Act.

However, neither clean air action planning nor noise reduction planning require consideration of peoples’ vulnerability or aspects of equity [68]. Currently, only existing environmental standards and the results of participation procedures are used as the basis for identifying points of intervention. In practice, this means that both planning instruments are likely to be implemented in areas showing higher levels of environmental burdens, particularly where residents do have the capacity to raise their concerns, but not necessarily in areas with higher shares of socioeconomically-disadvantaged residents. The results of the analysis shown above could be used as an additional criterion to target intervention areas and to prioritize suitable actions. 

Urban planning also provides health-promoting instruments, such as green space management. Existing green space management could focus on the identified neighborhoods of low green space availability, taking the additional burden of noise into account. The assignment of quiet areas is an element of noise action planning and could be merged with green space management, especially in those areas facing noise burden.

Furthermore, the Federal Building Code of Germany includes the “Social City” program, which pays particular attention to the built environment in deprived neighborhoods. To date, the identification and delineation of target areas has mainly been guided by social indicators. Using the results of the above analysis may help to strengthen health aspects in the selection of target areas [69]. As the program is institutionalized in the Federal Building Code, which demands not only a healthy urban development, but also that land use is socially just, it also provides a good basis for the integration of health and equity aspects.

## 5. Conclusions

The aim of this paper was to map socioeconomically-driven environmental inequalities relevant for health at the neighborhood level in order to identify options for concrete planning interventions. The analysis of the City of Dortmund revealed that various hotspots of environmental inequalities exist within the city. All selected socioeconomic indicators correlated quite strongly and helped to identify specific neighborhoods that house a significantly higher proportion of residents of a lower socio-economic position. We further found significant associations between the selected environmental indicators resulting in cumulative environmental burdens in various neighborhoods. Finally, we mapped levels of exposure by combining environmental state and social context indicators. The neighborhoods showing significant levels of multiple environmental burdens and at the same time housing a large proportion of socioeconomically-disadvantaged residents clearly demonstrate health-related environmental inequalities.

The toolbox of urban planning includes various planning instruments that help to address issues as identified above. In particular, instruments such as clean air action planning and noise reduction planning address environmental burdens as mapped here. However, in current planning practice, these instruments do not distinguish between populations of different socioeconomic positions, failing to recognize that those of lower socioeconomic position are more vulnerable and less able to cope with environmental burdens. Accordingly, the case for considering the vulnerability of the population in planning practice [62] might be strengthened based on the analysis carried out in this paper. 

Further research is needed to advance the study of health-related environmental inequalities into studying environmental justice issues, because this requires an additional analysis of the severity, consequences or morality of the inequalities [18]. This entails the evaluation of the magnitude of the uneven distribution of environmental burdens and amenities [15]. Another challenging research issue is the assessment of cumulative environmental impacts. Going beyond the pure overlaying or adding up of multiple burdens indicating a spatial and temporal concurrence necessitates, among other things, the assessment of combined and synergetic effects of multiple environmental stressors [70]. Finally, it would be interesting to study the impacts of proposed interventions in terms of reduced inequalities in the long run. Although intra-urban socioeconomic patterns are affected by various and even contradicting trends and developments from the sub-city to the national and European level, the indicator framework as developed in this study might as well be used for a long-term panel analysis for this purpose. 

## Figures and Tables

**Table 1:** GIS datasets.

Data Set	Year	Details
Noise impact areas: noise levels measured as annual average of 24 h noise emissions, in L_den_ db(A)	2013	Noise pollution data are modelled for five sources of noise emissions (train, tram, cars, industry, airport), using a noise dispersion model [44]
Ambient air quality: PM_10_ and NO_2_ measured as annual average emissions, in μg/m^3^	Data from 2008 to 2012, modeled in 2013	Emissions from various sources (transport, industry, housing) modelled in a 125 × 125-m grid system using the dispersion model [45]
Land use ^1^: current land use, mapped in 150 categories	2014	Mapped from aerial photographs at the 1:5000 scale

^1^ Data provided by Regionalverband Ruhr (RVR) [46].

**Table 2:** Census datasets.

Data Set	Year	Details
Total population	2013	Total number of inhabitants per neighborhood
Working population	2013	Total number of inhabitants per neighborhood between 15 and 65 (working age)
Unemployed population	2013	Total number of inhabitants per neighborhood receiving unemployment benefit subsistence (SGBII)
Population having a background of migration	2013	Total number of inhabitants per neighborhood having a background of migration (either themselves or at least one of their parents not being German)
Population receiving welfare aids	2014	Total number of inhabitants of non-working age per neighborhood receiving subsistence (welfare aids)

**Table 3:** Selected indicators.

Indicator	Details
Migration	Inhabitants having a background of migration as a % of the total population in the neighborhood
Unemployment	Inhabitants receiving unemployment benefits as a % of the total population between 18 and 65
Welfare	Inhabitants younger than 15 and older than 65 receiving social welfare aids as a % of the total population
Socioeconomic-Disadvantage	Sum of inhabitants receiving either unemployment benefits or social welfare aids as a % of the total population
Green	Share of green area (parks and forests), >1 ha, including green areas in a 400-m zone surrounding the neighborhood, as a % of the total area of the neighborhood
Noise	Share of area having a noise impact >55 dB(A) L_den_ as a % of the total area of the neighborhood
NO_2_	Share of area having an annual average value of NO_2_ larger than or equal to 30 μg/m³ as a % of the total area of the neighborhood
PM_10_	Share of area having an annual average value of PM_10_ larger than or equal to 25 μg/m³ as a % of the total area of the neighborhood

**Table 4:** Spearman rank correlation coefficients between social context indicators.

Indicators	Migration	Unemployment	Welfare	Socioeconomic Disadvantage
Migration	1.00000	0.85655 *	0.77527 *	0.86507 *
Unemployment	0.85655 *	1.00000	0.81576 *	0.98965 *
Welfare	0.77527 *	0.81576 *	1.00000	0.86939 *
Socioeconomic disadvantage	0.86507 *	0.98965 *	0.86939 *	1.00000

* *p*-value < 0.05.

**Table 5:** Spearman rank correlation coefficients between environmental indicators.

Indicators	Green	Noise	NO_2_	PM_10_
Green	1.00000	−0.15649 *	−0.23524 *	−0.17371 *
Noise	−0.15649 *	1.00000	0.42975 *	0.49360 *
NO_2_	−0.23524 *	0.42975 *	1.00000	0.81598 *
PM_10_	−0.17371 *	0.49360 *	0.81598 *	1.00000

* *p*-value < 0.05.

**Table 6:** Spearman rank correlation coefficients between neighborhood socioeconomic disadvantage and environmental indicators.

Indicator	Green	Noise	NO_2_	PM_10_
Socioeconomic disadvantage	−0.279 *	−0.01652	0.31980 *	0.25658 *

* *p*-value <0.05.

## Supplementary Materials

The following are available online at www.mdpi.com/www.mdpi.com/1660-4601/13/7/711/s1, Table S1. Original indicator values for 170 neighborhoods. Table S2. Reclassified indicators into quartiles.
