# Supplementary material for: Mapping Environmental Inequalities Relevant for Health for Informing Urban Planning Interventions—A Case Study in the City of Dortmund, Germany
Source: Int J Environ Res Public Health. 2016 Jul 13;13(7):711. doi: 10.3390/ijerph13070711 (PMC4962252; doi:10.3390/ijerph13070711)
Supplement: Supplementary file 1 [file ijerph-13-00711-s001.pdf]

Supplementary Materials: Mapping Environmental Inequalities Relevant for Health for Informing Urban Planning Interventions—A Case Study in the City of Dortmund, Germany

Johannes Flacke, Steffen Andreas Schüle, Heike Köckler and Gabriele Bolte

Table S1. Original indicator values for 170 neighborhoods.

Neighborhood	Name	Migration (%)	Unemployment (%)	Welfare (%)	Socioeconomic Disadvantage (%)	Green (%)	Noise (%)	NO_2 (%)	PM_{10} (%)
1	City-Ost	42.1	16.4	3.9	16.4	9.8	50.6	100.0	83.6
2	City-West	45.7	27.0	4.0	20.6	7.1	56.8	100.0	83.1
3	Cityring-West	27.7	16.1	1.4	14.2	18.8	60.6	100.0	79.7
4	Cityring-Ost	47.5	19.8	3.4	17.7	6.6	80.9	100.0	100.0
11	Westfalenhalle	15.5	5.2	1.1	4.8	29.6	72.9	99.5	71.0
12	Südwestfriedhof	16.3	6.4	0.7	5.3	51.3	88.8	100.0	84.0
13	Tremonia	19.9	9.8	1.8	8.2	24.5	76.9	100.0	35.4
21	Westpark	25.5	11.6	1.6	10.5	24.2	55.1	100.0	79.6
22	Dorstfelder Brücke	41.5	22.5	2.9	19.5	21.3	47.6	100.0	78.8
23	Union	61.6	31.2	3.2	26.2	6.9	94.3	100.0	81.9
31	Hallerey	47.9	25.4	3.2	19.8	19.6	91.0	80.5	60.6
32	Dorstfeld	47.7	28.0	2.4	20.0	19.3	87.0	100.0	58.4
33	Oberdorstfeld	20.7	12.0	1.2	9.2	13.8	90.3	100.0	61.9
41	Hafen	57.3	34.5	3.9	27.7	22.9	51.8	70.3	87.9
42	Hafen-Süd	58.7	34.8	2.8	27.6	4.5	81.2	100.0	98.5
43	Hafen-Südost	56.7	35.1	3.0	28.0	24.0	45.6	100.0	100.0
51	Nordmarkt-Süd	76.6	38.7	5.0	29.9	12.4	69.8	100.0	100.0
52	Nordmarkt-Südost	72.1	41.4	3.8	31.7	11.8	56.7	100.0	100.0
53	Nordmarkt-West	65.3	36.5	3.1	27.3	30.6	60.6	87.0	62.6
54	Nordmarkt-Ost	74.8	36.1	3.8	27.7	9.2	79.1	88.3	46.0
61	Borsigplatz	70.1	39.0	3.8	30.1	12.3	77.0	100.0	96.1
62	Westfalenhütte	67.7	36.4	2.6	26.8	3.3	74.2	24.6	12.6
71	Kaiserbrunnen	31.7	13.6	1.8	11.7	19.7	48.4	100.0	70.7
72	Funkenburg	37.9	21.0	3.8	18.8	17.6	42.6	100.0	37.9
73	Körne	33.2	16.6	1.8	12.5	10.4	51.6	92.4	23.6

81	Westfalendamm-Nord	23.2	8.2	0.8	5.8	18.5	80.0	100.0	60.7
82	Gartenstadt-Nord	27.7	16.1	1.5	10.8	13.2	86.9	100.0	47.8
83	Westfalendamm-Süd	23.2	8.7	0.9	6.2	36.0	64.8	97.9	52.8
84	Gartenstadt-Süd	13.9	2.1	0.4	1.6	12.7	98.4	100.0	40.6
91	Ruhrallee West	20.2	8.2	1.3	7.3	35.2	79.8	100.0	91.3
92	Ruhrallee Ost	23.4	8.2	1.1	6.8	52.0	84.4	99.7	67.3
111	Brechten-Nord	12.8	6.5	0.4	4.4	3.8	83.9	54.1	29.0
112	Brechten-Süd	14.7	9.7	0.6	6.7	44.8	84.5	71.3	32.1
121	Niedereving	50.5	27.8	1.5	18.4	50.0	36.7	31.8	71.5
122	Eving	32.3	23.7	1.9	16.0	46.0	48.7	29.4	20.2
123	Obereving	47.5	25.4	1.9	18.7	14.5	46.4	38.0	17.6
124	Kemminghausen	34.4	25.4	2.1	19.1	12.1	69.3	31.0	4.6
130	Holthausen	8.2	2.8	0.0	1.8	32.4	42.2	17.5	7.6
140	Lindenhorst	41.3	23.8	1.5	17.1	28.0	66.7	25.7	91.5
211	Altenderne	17.3	9.8	1.7	8.2	6.0	98.2	99.9	73.8
212	Derne	40.6	20.7	1.1	13.6	8.0	77.6	68.4	19.2
221	Grevel	15.6	3.4	0.0	2.2	21.4	16.9	0.7	0.0
222	Hostedde	17.9	9.4	1.1	7.2	17.3	61.4	20.0	1.3
231	Franz-Zimmer-Siedlung	37.9	16.9	0.8	10.6	6.1	86.8	74.2	35.3
232	Kirchderne	21.4	13.6	1.1	10.0	9.6	95.9	69.4	39.4
241	Kurl-Nord	8.6	6.7	0.4	4.4	66.2	91.6	1.3	12.6
242	Kurl-Süd	22.5	14.7	0.8	9.2	36.9	59.1	3.6	5.8
243	Husen-Nord	13.6	15.0	0.6	10.3	56.4	98.8	0.0	5.5
244	Husen-Süd	20.5	5.3	0.5	3.9	35.6	55.6	0.0	8.0
251	Lanstrop-Neu	29.0	25.0	2.9	18.5	9.1	81.2	55.3	18.3
252	Lanstrop-Alt	9.8	3.8	0.3	2.7	37.1	22.1	2.3	0.0
261	MSA-Siedlung	24.9	8.1	1.4	6.1	21.5	53.0	11.0	2.2
262	Alt-Scharnhorst	38.8	23.4	1.7	16.5	7.6	95.4	72.5	46.7
263	Westholz	20.4	9.4	0.8	6.1	13.8	92.7	26.6	14.9
270	Scharnhorst-Ost	63.6	35.5	3.3	23.9	32.0	66.2	5.8	4.7
311	Asselburg	14.2	10.9	1.2	7.9	8.8	18.1	12.4	0.7
312	Asseln Dorf	19.6	15.8	0.9	11.2	1.7	26.5	41.2	4.3
313	Asseln Hellweg	13.7	12.6	0.4	8.1	3.0	63.4	70.1	22.9
314	Kolonie Holstein	15.0	4.7	0.5	3.5	1.1	58.9	22.9	3.1
315	Kolonie Neuasseln	15.1	8.8	0.7	6.2	1.2	78.8	63.9	25.2
321	Brackeler Feld	19.4	5.1	0.4	3.6	27.2	58.6	21.3	14.7

322	Westheck	21.6	10.9	1.3	7.5	6.7	65.9	39.1	21.2
323	Reichshof	32.1	21.7	2.2	15.0	12.6	15.2	40.2	4.6
324	Brackel Dorf	17.5	7.5	0.7	5.1	6.4	15.6	37.5	6.8
325	Brackel Hellweg	14.5	8.6	0.3	5.9	17.7	60.1	74.8	36.4
326	Knappschaftkrankenhaus	21.6	8.6	1.4	7.0	48.0	46.4	71.6	27.8
327	Hauptfriedhof	30.9	13.5	3.7	11.8	67.3	77.8	68.2	20.9
328	Funkturmsiedlung	28.2	17.2	3.5	13.8	30.3	57.8	71.3	21.6
331	Wambel Dorf	22.4	12.4	1.6	9.6	3.2	80.4	87.6	37.3
332	Breierspfad	20.4	8.1	1.0	5.9	20.1	48.6	98.5	43.3
333	Pferderennbahn	34.7	3.4	0.7	3.0	49.5	80.6	89.5	43.2
341	Wickeder Feld	35.3	26.0	3.2	19.2	20.4	7.8	0.8	0.0
342	Wickede Dorf	21.5	13.6	1.1	9.4	11.2	34.2	38.0	14.4
343	Dollersweg	26.5	14.5	1.4	10.4	23.0	26.0	42.8	6.9
344	Flughafen	26.5	18.6	1.0	12.9	3.1	80.3	62.6	8.9
411	Aplerbecker Straße	21.0	8.5	0.7	5.7	19.3	99.3	100.0	38.7
412	Marsbruchstraße	25.8	14.4	2.2	11.0	48.5	84.2	100.0	34.3
413	Aplerbecker Markt	30.7	18.9	1.9	12.7	15.3	90.5	75.8	33.7
414	Vieselerhofstraße	18.8	8.0	0.9	5.7	15.4	81.6	34.2	16.4
415	Aplerbeck Bahnhof Süd	15.8	8.1	1.1	6.1	12.4	49.3	34.2	3.3
416	Aplerbecker Mark	11.6	1.8	0.3	1.3	50.2	22.2	23.2	5.1
417	Schwerter Straße	9.5	3.8	0.5	2.6	58.7	63.7	17.1	3.6
421	Berghofen Dorf	19.4	10.4	0.9	7.0	28.9	38.7	48.4	29.9
422	Ostkirchstraße	17.3	5.7	0.8	4.4	26.7	43.6	21.9	4.0
423	Berghofer Mark	9.6	1.9	0.2	1.4	42.1	21.2	14.8	4.9
431	Schüren-Neu	28.4	14.8	1.8	10.7	21.0	52.8	94.0	22.2
432	Schüren-Alt	22.7	11.1	1.1	8.3	16.4	65.8	89.2	37.4
441	Sölde-Nord	15.4	10.6	1.6	7.9	7.0	99.0	76.3	38.4
442	Sölde-Süd	15.1	9.4	1.1	7.0	28.0	97.8	30.6	67.6
451	Sölderholz	8.5	3.5	0.6	2.6	35.0	78.1	32.1	17.3
452	Lichtendorf	10.0	3.6	0.5	2.7	27.7	85.4	77.9	45.7
511	Benninghofen	21.7	15.0	2.6	11.3	23.6	28.1	42.5	21.2
512	Loh	12.4	3.2	0.4	2.3	19.4	19.0	13.6	1.2
521	Schulzentrum Hacheney	34.9	17.7	0.6	12.4	64.9	79.2	61.8	41.6
522	Pferdebachtal	13.9	5.6	2.1	5.3	36.1	82.2	69.7	36.1
531	Remberg	32.6	20.3	3.2	16.6	34.5	56.6	94.0	15.6
532	Hörde	39.0	25.5	4.2	21.0	27.2	43.4	93.7	31.1

533	Phönix-West	32.0	32.5	4.4	27.1	45.3	57.3	55.6	19.0
534	Brücherhof	29.2	23.0	3.1	18.5	26.6	30.0	56.0	24.5
535	Clarenberg	66.5	45.2	10.6	36.6	30.0	84.6	91.2	61.2
541	Höchsten	11.5	1.9	0.6	1.7	48.1	20.6	11.3	1.5
542	Holzen	15.6	5.2	0.8	3.9	30.3	87.1	78.9	27.8
551	Syburg	13.7	1.6	0.6	1.6	60.7	38.0	20.6	7.5
552	Buchholz	9.8	1.2	0.0	0.8	68.7	51.9	31.1	13.0
561	Wellinghofen	20.8	9.8	1.4	7.5	25.0	44.7	49.1	24.3
562	Durchstraße	13.6	4.9	0.6	3.4	25.3	35.3	15.5	9.1
570	Wichlinghofen	8.6	1.7	0.2	1.3	59.6	55.3	33.0	15.7
611	Schönau	14.5	2.9	0.3	2.2	10.7	85.8	100.0	45.1
612	Ostenbergstraße	16.2	3.8	0.0	2.5	36.9	51.5	100.0	14.8
613	Krückenweg	25.8	11.5	1.2	8.7	28.9	43.9	66.4	19.7
614	Baroper Markt	20.7	10.4	1.4	8.3	34.3	36.8	66.0	24.6
615	Zechenplatz	35.0	5.6	2.1	5.7	8.6	78.8	95.5	56.7
620	Bittermark	14.9	3.6	0.5	2.7	72.3	87.2	42.5	18.9
631	Brünninghausen	10.5	1.5	0.0	0.9	57.0	33.3	39.4	11.8
632	Renninghausen	17.7	11.7	1.7	7.9	35.8	55.8	75.7	38.1
641	Eichlinghofen	19.0	4.8	0.4	3.6	31.6	75.5	86.2	30.4
642	Universität	36.0	1.8	0.4	1.9	28.3	68.1	95.7	28.4
643	Salingen	14.8	2.4	0.0	1.6	20.1	98.8	98.1	16.1
651	Hombruch	24.0	15.8	2.6	13.1	15.7	44.4	48.9	5.2
652	Luxemburger-Straße	21.3	11.1	1.6	8.5	12.1	25.9	14.0	3.3
653	Rotkehlchenweg	24.6	15.1	0.4	8.8	23.7	24.7	27.0	10.9
661	Persebeck	16.6	8.4	0.3	5.3	8.7	99.2	89.1	38.4
662	Kruckel	21.8	4.4	0.5	3.2	12.6	98.8	82.8	17.0
663	Schnee	9.9	2.2	0.0	1.4	50.3	72.0	20.5	6.3
671	Großholthausen	10.3	2.9	0.0	1.7	27.6	75.8	43.4	17.2
672	Kirchhörde-Nord	30.0	18.8	1.3	12.1	21.2	24.9	0.0	0.0
673	Kleinholthausen	13.9	1.6	0.0	0.9	29.2	26.3	18.5	5.9
674	Kirchhörde-Ost	11.1	2.1	0.5	1.7	38.2	18.3	4.2	0.0
675	Kirchhörde-West	12.2	4.2	0.7	3.1	51.8	51.5	34.6	18.6
676	Löttringhausen-Nord	18.8	10.5	0.7	6.3	52.8	59.9	3.0	0.0
677	Löttringhausen-Süd	14.0	6.3	1.0	4.9	58.4	87.1	38.3	14.7
678	Schanze	14.8	6.0	0.0	3.8	72.4	96.4	37.0	8.0
681	Rombergpark	17.8	0.0	1.3	1.3	75.4	54.9	37.4	18.9

682	Lücklemberg	13.6	2.2	0.3	1.5	45.9	41.3	23.3	8.6
690	Menglinghausen	25.7	11.7	2.0	9.5	19.7	57.5	52.5	7.9
710	Bövinghausen	28.8	26.8	2.4	19.4	30.2	6.8	5.7	0.0
720	Kley	17.4	12.6	0.8	9.1	21.4	63.1	52.4	17.7
731	Holte-Kreta	16.3	9.7	1.5	8.0	21.6	6.8	0.0	0.0
732	Deipenbeck	17.2	11.8	1.1	8.6	45.3	32.8	30.4	6.0
733	Lütgendortmund-Mitte	26.3	18.8	1.4	13.2	37.8	43.5	51.8	28.0
734	Lütgendortmund-Ost	26.3	17.7	1.8	13.8	15.2	83.7	96.9	65.2
735	Lütgendortmund-West	41.7	16.8	1.6	12.4	22.2	85.5	98.7	63.2
736	Somborn	11.4	10.5	0.9	8.2	21.7	99.3	78.5	61.1
741	Germania	32.2	26.8	2.8	20.7	13.0	79.2	91.5	26.8
742	Marten	25.4	19.9	1.8	15.0	17.7	97.7	100.0	83.0
750	Oespel	16.0	7.2	0.7	5.3	24.5	83.0	84.7	46.2
760	Westrich	16.3	7.0	0.8	5.3	17.9	13.6	1.5	0.0
810	Deusen	18.9	7.9	0.4	5.7	5.0	75.3	22.4	75.0
821	Mailoh	39.1	21.3	2.2	15.9	29.0	59.2	16.2	31.2
822	Erpinghofsiedlung	39.2	25.5	3.1	20.1	67.1	26.1	3.5	1.5
823	Wischlingen	29.4	11.7	1.4	8.6	44.5	98.5	98.5	38.6
824	Huckarde	33.2	23.0	1.8	16.5	22.2	55.1	90.2	64.0
825	Insterburgsiedlung	33.9	15.5	1.6	11.3	6.9	60.3	100.0	71.7
831	Jungferntal	25.3	19.0	1.5	13.0	47.4	62.9	51.7	24.3
832	Rahm	16.8	7.7	0.8	5.6	36.5	99.8	100.0	64.0
841	Kirchlinde-Alt	28.0	21.1	1.3	14.8	16.4	86.8	69.9	28.1
842	Siedlung Siepmannstraße	39.2	27.1	2.4	18.4	22.7	11.3	7.6	0.5
843	Hangeney	22.3	14.1	0.7	9.7	15.9	47.8	53.8	29.6
910	Bodelschwingh	33.2	13.8	2.1	11.0	25.0	70.7	32.7	12.9
921	Brüninghausen/Knepper	24.7	10.7	0.0	6.7	35.5	95.1	27.3	52.5
922	Mengeder Heide	11.9	4.7	0.6	3.6	33.5	99.7	75.8	51.2
923	Mengede-Mitte	27.7	16.4	1.3	11.2	14.3	83.7	32.3	26.4
924	Alte Kolonie	37.5	24.8	1.6	18.2	16.3	95.2	15.3	12.2
930	Nette	35.7	26.4	2.4	18.6	24.3	63.4	10.7	4.2
940	Oestrich	32.4	18.9	1.7	14.1	5.8	91.1	48.6	34.0
951	Groppenbruch	18.8	2.1	0.0	1.3	3.5	77.3	48.9	27.3
952	Schwieringhausen	11.2	9.4	0.0	6.3	20.4	98.5	60.8	22.5
953	Ellinghausen	9.6	4.9	3.4	7.0	1.9	44.1	17.3	7.1
954	Niedernette	2.7	0.0	0.0	0.0	2.7	93.8	12.2	2.5

960	Westerfilde	41.8	34.0	2.2	22.7	49.7	59.1	30.5	14.4

Table S2. Reclassified indicators into quartiles.

Neighborhood	Name	Area (m^2)	Unemployment	Socioeconomic Disadvantage	Green	Noise	NO_2	PM_{10}
1	City-Ost	514,115.3	3	4	1	2	4	4
2	City-West	426,586.3	4	4	1	2	4	4
3	Cityring-West	608,571.2	3	3	2	2	4	4
4	Cityring-Ost	180,741.6	3	4	1	3	4	4
11	Westfalenhalle	2,009,518.7	1	1	3	3	4	4
12	Südwestfriedhof	429,671.7	2	2	4	4	4	4
13	Tremonia	1,300,825.6	2	2	3	3	4	3
21	Westpark	382,697.2	3	3	3	2	4	4
22	Dorstfelder Brücke	399,994.8	4	4	2	2	4	4
23	Union	1,550,716.7	4	4	1	4	4	4
31	Hallerey	2,150,140.9	4	4	2	4	3	4
32	Dorstfeld	1,553,517.7	4	4	2	4	4	4
33	Oberdorstfeld	2,552,666.4	3	3	2	4	4	4
41	Hafen	3,272,649.5	4	4	3	2	3	4
42	Hafen-Süd	1,039,881.1	4	4	1	3	4	4
43	Hafen-Südost	277,372.3	4	4	3	1	4	4
51	Nordmarkt-Süd	637,511.1	4	4	1	3	4	4
52	Nordmarkt-Südost	131,130.2	4	4	1	2	4	4
53	Nordmarkt-West	1,129,676.0	4	4	3	2	3	4
54	Nordmarkt-Ost	1,311,068.4	4	4	1	3	3	3
61	Borsigplatz	509,516.0	4	4	1	3	4	4
62	Westfalenhütte	6,075,540.5	4	4	1	3	1	2
71	Kaiserbrunnen	889,082.0	3	3	2	2	4	4
72	Funkenburg	1,120,935.1	4	4	2	1	4	3
73	Körne	1,780,022.0	3	3	1	2	3	2
81	Westfalendamm-Nord	1,263,193.8	2	2	2	3	4	4
82	Gartenstadt-Nord	1,422,714.7	3	3	2	4	4	4
83	Westfalendamm-Süd	873,871.1	2	2	4	3	4	4
84	Gartenstadt-Süd	1,025,203.9	1	1	2	4	4	3
91	Ruhrallee West	1,297,581.2	2	2	3	3	4	4
92	Ruhrallee Ost	1,634,928.7	2	2	4	4	4	4
111	Brechten-Nord	4,718,645.9	2	1	1	3	2	3

112	Brechten-Süd	2,500,883.3	2	2	4	4	3	3
121	Niedereving	854,095.8	4	4	4	1	2	4
122	Eving	2,665,169.3	4	4	4	2	2	2
123	Obereving	2,039,927.4	4	4	2	2	2	2
124	Kemminghausen	1,943,434.1	4	4	1	3	2	1
130	Holthausen	4,957,240.5	1	1	3	1	1	1
140	Lindenhorst	3,317,536.5	4	4	3	3	1	4
211	Altenderne	1,878,786.5	2	2	1	4	4	4
212	Derne	2,050,215.8	4	3	1	3	3	2
221	Grevel	2,288,579.9	1	1	2	1	1	1
222	Hostedde	2,028,250.4	2	2	2	2	1	1
231	Franz-Zimmer-Siedlung	1,365,041.5	3	3	1	4	3	3
232	Kirchderne	1,233,675.0	3	3	1	4	3	3
241	Kurl-Nord	1,243,042.4	2	1	4	4	1	2
242	Kurl-Süd	2,800,815.2	3	3	4	2	1	1
243	Husen-Nord	452,877.5	3	3	4	4	1	1
244	Husen-Süd	1,714,390.0	1	1	3	2	1	1
251	Lanstrop-Neu	2,445,006.1	4	4	1	3	2	2
252	Lanstrop-Alt	4,735,772.1	1	1	4	1	1	1
261	MSA-Siedlung	1,546,503.5	2	2	2	2	1	1
262	Alt-Scharnhorst	1,958,791.7	4	4	1	4	3	4
263	Westholz	1,058,014.3	2	2	2	4	1	2
270	Scharnhorst-Ost	2,834,078.9	4	4	3	3	1	1
311	Asselburg	2,268,290.0	2	2	1	1	1	1
312	Asseln Dorf	439,934.5	3	3	1	1	2	1
313	Asseln Hellweg	485,964.8	3	2	1	2	3	2
314	Kolonie Holstein	3,350,570.3	1	1	1	2	1	1
315	Kolonie Neuasseln	1,696,512.9	2	2	1	3	3	3
321	Brackeler Feld	3,302,482.7	1	1	3	2	1	2
322	Westheck	1,403,131.5	2	2	1	3	2	2
323	Reichshof	388,259.3	4	4	1	1	2	1
324	Brackel Dorf	679,209.7	2	2	1	1	2	1
325	Brackel Hellweg	465,362.3	2	2	2	2	3	3
326	Knappschaftkrankenhaus	535,386.2	2	2	4	2	3	3
327	Hauptfriedhof	2,053,345.6	3	3	4	3	3	2
328	Funkturmsiedlung	1,024,068.9	3	3	3	2	3	2

331	Wambel Dorf	1,604,303.7	3	3	1	3	3	3
332	Breierspfad	760,614.4	2	2	2	2	4	3
333	Pferderennbahn	1,127,611.8	1	1	4	3	3	3
341	Wickeder Feld	3,341,056.3	4	4	2	1	1	1
342	Wickede Dorf	814,294.6	3	3	1	1	2	2
343	Dollersweg	523,740.6	3	3	3	1	2	1
344	Flughafen	4,290,268.1	3	3	1	3	3	2
411	Aplerbecker Straße	1,255,313.6	2	2	2	4	4	3
412	Marsbruchstraße	1,105,965.8	3	3	4	3	4	3
413	Aplerbecker Markt	945,330.7	3	3	2	4	3	3
414	Vieselerhofstraße	990,239.9	2	2	2	3	2	2
415	Aplerbeck Bahnhof Süd	1,034,042.5	2	2	1	2	2	1
416	Aplerbecker Mark	1,726,573.6	1	1	4	1	1	1
417	Schwerter Straße	1,677,933.5	1	1	4	3	1	1
421	Berghofen Dorf	1,931,509.0	2	2	3	1	2	3
422	Ostkirchstraße	992,307.6	1	1	3	1	1	1
423	Berghofer Mark	2,199,303.2	1	1	4	1	1	1
431	Schüren-Neu	1,987,414.7	3	3	2	2	4	2
432	Schüren-Alt	1,961,930.4	2	3	2	3	3	3
441	Sölde-Nord	1,936,222.4	2	2	1	4	3	3
442	Sölde-Süd	519,498.1	2	2	3	4	2	4
451	Sölderholz	2,984,177.5	1	1	3	3	2	2
452	Lichtendorf	1,916,139.8	1	1	3	4	3	3
511	Benninghofen	1,077,459.9	3	3	3	1	2	2
512	Loh	1,570,248.6	1	1	2	1	1	1
521	Schulzentrum Hacheney	609,238.4	3	3	4	3	3	3
522	Pferdebachtal	706,325.8	1	2	4	3	3	3
531	Remberg	1,439,153.5	4	4	3	2	4	2
532	Hörde	1,478,983.4	4	4	3	1	4	3
533	Phönix-West	1,547,003.1	4	4	4	2	3	2
534	Brücherhof	639,877.6	4	4	3	1	3	3
535	Clarenberg	756,777.3	4	4	3	4	3	4
541	Höchsten	2,504,436.2	1	1	4	1	1	1
542	Holzen	5,504,288.8	1	1	3	4	3	3
551	Syburg	6,193,285.4	1	1	4	1	1	1
552	Buchholz	1,905,188.6	1	1	4	2	2	2

561	Wellinghofen	1,034,472.8	2	2	3	1	2	3
562	Durchstraße	992,121.3	1	1	3	1	1	2
570	Wichlinghofen	1,797,823.5	1	1	4	2	2	2
611	Schönau	424,415.7	1	1	1	4	4	3
612	Ostenbergstraße	182,061.3	1	1	4	2	4	2
613	Krückenweg	897,141.5	3	3	3	1	3	2
614	Baroper Markt	289,845.5	2	2	3	1	3	3
615	Zechenplatz	186,317.4	1	2	1	3	4	4
620	Bittermark	3,563,821.4	1	1	4	4	2	2
631	Brünninghausen	1,290,418.5	1	1	4	1	2	2
632	Renninghausen	902,431.7	3	2	4	2	3	3
641	Eichlinghofen	2,491,537.5	1	1	3	3	3	3
642	Universität	2,827,056.9	1	1	3	3	4	3
643	Salingen	1,264,220.9	1	1	2	4	4	2
651	Hombruch	768,090.1	3	3	2	1	2	1
652	Luxemburger-Straße	1,172,011.1	2	3	1	1	1	1
653	Rotkehlchenweg	292,334.0	3	3	3	1	2	2
661	Persebeck	2,024,447.0	2	2	1	4	3	3
662	Kruckel	678,736.3	1	1	2	4	3	2
663	Schnee	2,483,869.0	1	1	4	3	1	1
671	Großholthausen	2,126,198.2	1	1	3	3	2	2
672	Kirchhörde-Nord	706,075.3	3	3	2	1	1	1
673	Kleinholthausen	380,036.2	1	1	3	1	1	1
674	Kirchhörde-Ost	729,523.9	1	1	4	1	1	1
675	Kirchhörde-West	1,407,916.6	1	1	4	2	2	2
676	Löttringhausen-Nord	375,520.2	2	2	4	2	1	1
677	Löttringhausen-Süd	1,050,716.7	2	2	4	4	2	2
678	Schanze	853,693.3	2	1	4	4	2	2
681	Rombergpark	1,498,610.9	1	1	4	2	2	2
682	Lücklemberg	2,085,399.6	1	1	4	1	1	2
690	Menglinghausen	1,990,818.3	3	3	2	2	2	1
710	Bövinghausen	2,561,890.0	4	4	3	1	1	1
720	Kley	1,571,806.6	3	3	2	2	2	2
731	Holte-Kreta	856,226.9	2	2	2	1	1	1
732	Deipenbeck	1,319,488.6	3	3	4	1	2	1
733	Lütgendortmund-Mitte	1,462,849.8	3	3	4	1	2	3

734	Lütgendortmund-Ost	1,915,187.9	3	3	2	3	4	4
735	Lütgendortmund-West	593,437.9	3	3	2	4	4	4
736	Somborn	962,749.4	2	2	2	4	3	4
741	Germania	1,210,769.6	4	4	2	3	3	3
742	Marten	3,068,590.4	4	4	2	4	4	4
750	Oespel	4,164,612.2	2	2	3	3	3	4
760	Westrich	2,679,124.5	2	2	2	1	1	1
810	Deusen	3,046,303.7	2	2	1	3	1	4
821	Mailoh	2,688,595.3	4	4	3	2	1	3
822	Erpinghofsiedlung	1,299,023.3	4	4	4	1	1	1
823	Wischlingen	1,009,531.9	3	3	4	4	4	3
824	Huckarde	617,214.1	4	4	2	2	3	4
825	Insterburgsiedlung	445,750.6	3	3	1	2	4	4
831	Jungferntal	1,069,937.2	3	3	4	2	2	2
832	Rahm	971,630.1	2	2	4	4	4	4
841	Kirchlinde-Alt	1,438,262.8	4	4	2	4	3	3
842	Siedlung Siepmannstraße	490,076.6	4	4	3	1	1	1
843	Hangeney	1,612,556.1	3	3	2	2	2	3
910	Bodelschwingh	3,294,080.8	3	3	3	3	2	2
921	Brüninghausen/Knepper	1,919,705.8	2	2	3	4	2	4
922	Mengeder Heide	2,382,104.9	1	1	3	4	3	4
923	Mengede-Mitte	1,720,029.1	3	3	2	3	2	3
924	Alte Kolonie	697,180.7	4	4	2	4	1	2
930	Nette	2,745,523.9	4	4	3	3	1	1
940	Oestrich	2,700,453.3	3	3	1	4	2	3
951	Groppenbruch	2,568,720.7	1	1	1	3	2	3
952	Schwieringhausen	1,062,069.3	2	2	2	4	3	2
953	Ellinghausen	4,726,413.0	1	2	1	1	1	1
954	Niedernette	1,873,529.2	1	1	1	4	1	1
960	Westerfilde	3,080,182.9	4	4	4	2	2	2

© 2016 by the authors; licensee MDPI, Basel, Switzerland. This article is an open access article distributed under the terms and conditions of the Creative Commons Attribution (CC-BY) license (http://creativecommons.org/licenses/by/4.0/).
